# Biochemical properties of a new thermo- and solvent-stable xylanase recovered using three phase partitioning from the extract of *Bacillus oceanisediminis* strain SJ3

**DOI:** 10.1186/s40643-017-0161-9

**Published:** 2017-07-05

**Authors:** Nawel Boucherba, Mohammed Gagaoua, Amel Bouanane-Darenfed, Cilia Bouiche, Khelifa Bouacem, Mohamed Yacine Kerbous, Yacine Maafa, Said Benallaoua

**Affiliations:** 1 Laboratory of Applied Microbiology, Faculty of Nature Science and Life, University of Bejaia, 06000 Bejaia, Algeria; 2 INATAA, Université des Frères Mentouri Constantine 1, Route de Ain El-Bey, 25000 Constantine, Algeria; 3 UMR1213 Herbivores, INRA, VetAgro Sup, Clermont Université, Université de Lyon, 63122 Saint-Genès-Champanelle, France; 4 Laboratory of Cellular and Molecular Biology, Microbiology Team, Faculty of Biological Sciences, University of Sciences, Technology of Houari Boumediene (USTHB), PO Box 32, El Alia, Bab Ezzouar, 16111 Algiers, Algeria

**Keywords:** *Bacillus oceanisediminis*, Xylanase, Thermostability, Hydrophobic solvents, Industrial processes, Three phase partitioning

## Abstract

The present study investigates the production and partial biochemical characterization of an extracellular thermostable xylanase from the *Bacillus oceanisediminis* strain SJ3 newly recovered from Algerian soil using three phase partitioning (TPP). The maximum xylanase activity recorded after 2 days of incubation at 37 °C was 20.24 U/ml in the presence of oat spelt xylan. The results indicated that the enzyme recovered in the middle phase of TPP system using the optimum parameters were determined as 50% ammonium sulfate saturation with 1.0:1.5 ratio of crude extract: *t*-butanol at pH and temperature of 8.0 and 10 °C, respectively. The xylanase was recovered with 3.48 purification fold and 107% activity recovery. The enzyme was optimally active at pH 7.0 and was stable over a broad pH range of 5.0–10. The optimum temperature for xylanase activity was 55 °C and the half-life time at this temperature was of 6 h. At this time point the enzyme retained 50% of its activity after incubation for 2 h at 95 °C. The crude enzyme resist to sodium dodecyl sulfate and β-mercaptoethanol, while all the tested ions do not affect the activity of the enzyme. The recovered enzyme is, at least, stable in tested organic solvents except in propanol where a reduction of 46.5% was observed. Further, the stability of the xylanase was higher in hydrophobic solvents where a maximum stability was observed with cyclohexane. These properties make this enzyme to be highly thermostable and may be suggested as a potential candidate for application in some industrial processes. To the best of our knowledge, this is the first report of xylanase activity and recoverey using three phase partitioning from *B. oceanisediminis.*

## Background

Hemicellulose is the second most abundant renewable biomass after cellulose in nature (Collins et al. 2005). Xylan is the major component of hemicelluloses in wood from angiosperms, where it accounts for 15–30% of the total dry weight. In gymnosperms, however, xylans contribute only 7–12% of the total dry weight. The structure of xylan is complex, and its complete biodegradation requires the concerted action of xylanolytic enzymes (Trajano et al. 2014; Zhang and Viikari 2014). Xylans are heterogeneous polysaccharides with a backbone consisting of β-1,4 linked d-xylosyl residues.

Endo-β-1,4 xylanases (EC 3.2.1.8) are the main enzymes responsible for cleavage of the linkages within the xylan backbone (Collins et al. 2005), to which short side chains of *O*-acetyl, α-l-arabinofuranosyl, d-α glucuronic, and phenolic acid residues are attached (Collins et al. 2005; Terrasan et al. 2010; Xie et al. 2015). Xylanases have been used in a wide range of industrial applications and processes. They have been applied in the bioconversion of lignocellulosic material and agro-wastes to fermentative products, clarification of juices, improvement in consistency of beer, and the digestibility of animal feed stock (Badhan et al. 2007; Elgharbi et al. 2015a, b; Shameer 2016; Jain and Krishnan 2017). Due to their important activity at alkaline pH (8.0–11) and high temperature (50–90 °C), thermostable alkaline xylanases have attracted special attention in the pulp bio-bleaching industry (Techapun et al. 2003; Bouacem et al. 2014; Boucherba et al. 2014; Bouanane-Darenfed et al. 2016). Xylanase, together with other hydrolytic enzymes, have also proved useful for the generation of bio-fuels, including ethanol, from lignocellulosic biomass. Xylanases are used in pulp pre-bleaching process to remove the hemicelluloses, which bind to the pulp. The hydrolysis of pulp bound hemicelluloses releases the lignin in the pulp, reducing the amount of chlorine required for conventional chemical bleaching and minimizing the toxic, chloroorganic waste. Therefore, xylanases from alkalophilic bacteria and actinomycetes and fungi have been studied widely (Perez-Rodriguez et al. 2014; Wang et al. 2014). However, large scale cultivation of fungi and actinomycetes is often difficult because of their slow generation time, coproduction of highly viscous polymers, and poor oxygen transfer (Wong et al. 1997; Garg et al. 2011). *Bacillus* genus is used more extensively than other bacteria in industrial fermentations, since they produce most of their enzymes. Some *Bacillus* strains have been reported as xylanolytic enzymes producers (Lindner et al. 1994; Seo et al. 2013; Tarayre et al. 2013; Elgharbi et al. 2015a; Zouari et al. 2015).

*Bacillus oceanisediminis* sp. nov. was first isolated from a marine sediment collected in the South Sea of China (Zhang et al. 2010). Considering the above, the present study was undertaken to described, for the first time, the production of a thermostable xylanase from *B. oceanisediminis* strain SJ3 recently isolated by our laboratory from Algerian soil, an attempt was made to biochemically characterize the xylanase activity secreted by this strain. Also, preliminary investigation using three phase partitioning (TPP) system (Gagaoua et al. 2014; Gagaoua and Hafid 2016) for xylanase purification was performed. In TPP process, firstly an inorganic salt (generally ammonium sulfate) is added to the crude extract containing proteins then mixted with *tert*-butanol in an appropriate amount (Gagaoua et al. 2015, 2016, 2017). When *t*-butanol is added in the presence of ammonium sulfate, it pushes the protein out of the solution. In this process *t*-butanol binds to hydrophobic part of the proteins to reduce the density of the proteins, leading to float above the denser aqueous salt phase. Within approximately an hour, it forms an interfacial (middle) precipitate between the lower aqueous and upper organic phase that usually contains *t*-butanol (Gagaoua and Hafid 2016).

## Methods

### Substrates, reagents, and chemicals

Birchwood xylan, oat spelt xylan, starch, carboxymethyl cellulose (CMC, low viscosity), *tert*-butanol, ammonium sulfate, and 3,5-dinitrosalicylic acid (DNS) were purchased from Sigma Chemical Company (St. Louis, MO, USA). Unless otherwise specified, all other reagents and chemicals were of the analytical grade or highest level of purity available.

### Collection of samples and culture conditions of microorganisms

The garden soil samples were collected from Bejaia north east of Algeria (Kabylia region) in March 2015. The soil was collected from five places and samples were pooled. Sub-samples of approximately 1 g were suspended in 100 ml sterile distilled water. Mixtures were allowed to settle and serial dilutions were prepared. From each dilution, 0.1 ml was taken and spread on agar plates of medium containing in g/l oat spelt xylan 10, yeast extract 2, NaCl 2.5, NH_4_Cl 5, KH_2_PO_4_ 15, Na_2_HPO_4_ 30, MgSO_4_·7H_2_O 0.25, and bacteriological agar 15. In this medium, there is a little modification of the main carbon source, the oat spelt xylan was used instead the birchwood xylan (Viet et al. 1991). The plates were incubated at pH 7 and 37 °C for 2 days at 250 rpm. Those colonies that grew well under such conditions and showed an orange zone around the colonies after red Congo were retained for second screening. Colonies with a clear zone formation following the hydrolysis of xylan were evaluated as xylanase producers. Several xylanlolytic strains were isolated and SJ3, which exhibited a large clear zone of hydrolysis, was selected and retained for further experimental study.

### Bacterial identification of the isolate SJ3

Analytical profile index (API) strip tests and 16S rRNA gene sequencing were carried out for the identification of the genus to which the strain belong.

API 50 CHB/E and the API 20E strips (bioMérieux, SA, Marcy-l’Etoile, France) were used to investigate the physiological and biochemical characteristics of strain SJ3, as recommended elsewhere (Logan and Berkeley 1984). The growth temperature (4, 10, 15, 20, 25, 30, 35, 40, and 45 °C), pH level values (4, 5, 6, 7, 8, 9, 10, 11, and 12) and sodium chloride regimes were determined.

The 16S rRNA gene was amplified by PCR using forward primer F-d1 5′-AGAGTTTGATCCTGGCTCAG-3′, and reverse primer R-d1 5′-AAGGAGGTGATCCAAGCC-3′, designed from base positions 8–27 and 1541–1525, respectively, which were the conserved zones within the rRNA operon of *Escherichia coli* (Gurtler and Stanisich 1996). The genomic DNA of strain SJ3 was purified using the Wizard^®^ Genomic DNA Purification Kit (Promega, Madison, WI, USA) and then used as a template for PCR amplification (30 cycles, 94 °C for 45 s denaturation, 60 °C for 45 s primer annealing, and 72 °C for 60 s extension). The amplified ~1.5 kb PCR product was cloned in the pGEM-T Easy vector (Promega, Madison, WI, USA), leading to pSJ3-16S plasmid (this study). The *E. coli* DH5α (F^−^ *supE44 Φ80 δlacZ ΔM15 Δ(lacZYA* **-** *argF) U169 endA1 recA1 hsdR17 (r*_*k*_^*−*^ *, m*_*k*_^+^ *) deoR thi* **-** *1 λ* ^−^ *gyrA96 relA1*) (Invitrogen, Carlsbad, CA, USA) was used as a host strain. All recombinant clones of *E. coli* were grown in Luria–Bertani (LB) broth media with the addition of ampicillin, isopropyl**-**thio**-**β**-** d **-**galactopyranoside (IPTG), and X-gal for screening. DNA electrophoresis, DNA purification, restriction, ligation, and transformation were all performed according to the method previously described elsewhere (Sambrook et al. 1989).

### DNA sequencing and molecular phylogenetic analysis

The nucleotide sequences of the cloned 16S rRNA gene were determined on both strands using BigDye Terminator Cycle Sequencing Ready Reaction kits and the automated DNA sequencer ABI PRISM^®^ 3100**-**Avant Genetic Analyser (Applied Biosystems, Foster City, CA, USA. The RapidSeq36_POP6 run module was used, and the samples were analyzed using the ABI sequencing analysis software v. 3.7 NT.

The sequences obtained were compared to those present in the public sequence databases and with the EzTaxon-e server (http://eztaxon-e.ezbiocloud.net/), a web-based tool for the identification of prokaryotes based on 16S rRNA gene sequences from type strains (Kim et al. 2012).

Phylogenetic and molecular evolutionary genetic analyses were conducted via the the molecular evolutionary genetics analysis (MEGA) software version 5 (http://www.megasoftware.net). Distances and clustering were calculated using the neighbor-joining method. The tree topology of the neighbor-joining data was evaluated by Bootstrap analysis with 100 re-samplings.

### Xylanase assay

Xylanase activity was determined by measuring the release of reducing sugar from soluble xylan using the DNS method (Miller 1959). In brief, 0.9 ml buffer A (10 mg/ml oat spelt xylan in 50 mM sodium-phosphate buffer at pH 7) were mixed with 0.1 ml of the recovered enzyme solution (1 mg/ml). After incubation at 55 °C for 10 min, the reaction was terminated by adding 1.5 ml of the DNS reagent (Maalej et al. 2009). The mixture was then boiled for 5 min and cooled. Absorption was measured at 540 nm.

One unit of xylanase activity was defined as the amount of enzyme that released 1 µmol of reducing sugar equivalent to xylose per min under the assay conditions.

### Xylanase production

#### Gowth condition of the xylanase activity

To study the properties of the xylanase activity production, the isolates having high xylanase activities were cultivated in 250 ml shake-flasks containing 50 ml basic xylanase production medium at 37 °C. The basic xylanase production medium was prepared at pH 7.0 containing oat spelt xylan. The culture was harvested after 48 h, and centrifuged (10,000 rpm for 10 min). Growth was measured by determining absorbance at 600 nm. The sample was then kept at 4 °C in the refrigerator.

#### Effect of incubation time on xylanase production

Pre-culture (2%) was used to inoculate 250 ml xylan defined medium at 37 °C for 72 h. culture samples were collected each 4 h during the cultivation period. Immediately after collection, the samples were centrifuged at 4 °C and 10,000*g* for 20 min. Supernatants were analyzed for xylanase activity as described above.

### Partial biochemical characterization of the recovered enzyme by TPP

#### Extraction and partial purification of xylanase by TPP

Aqueous systems such as three phase partitioning (TPP), known as simple, economical and quick methods, were described for the fast recovery of enzymes (Gagaoua and Hafid 2016). This elegant non-chromatographic tool may be performed in a purification process to be used successfully in food or other industries. For its application in this study, the crude extract was first collected after 48 h of batch incubation (Boucherba et al. 2014). The culture supernatant containing secreted xylanases was concentrated using Sartorius membranes (with 10-kDa cutoff membrane; Millipore) after a centrifugation at 10,000 rpm for 10 min. Then, TPP experiments were carried out following the recommendations of Gagaoua et al. (2015). The enzyme exclusively recovered in the interfacial phase was gently separated from the other phases and dissolved in 50 mM Tris–HCl buffer (pH 8.5) and dialyzed overnight at 4–5 °C and used for enzyme characterization.

#### Effect of temperature and pH on xylanase activity

Optimal temperature was determined by assaying the enzyme activity between 20 and 100 °C, by incubating the enzyme along with the substrate for 10 min at the respective temperature. Relative xylanase activity was determined using 10 mg/ml oat spelt xylan at various pHs. The pH range used varied from 4 to 10. Three different buffers (50 mM) were used. Sodium acetate buffer was used for pH 4–6; Sodium-phosphate buffer was used for pH from 6 to 7 and Tris–HCl buffer for pH 7–10.

#### Effect of temperature on xylanase stability

The thermostability was determined at temperatures of 50, 55, 60, and 95 °C, after incubation with the substrate for different times (from 0.5 to 7 h); remaining xylanase activity was measured under standard assay conditions. The non-heated enzyme, which was left at room temperature, was considered as control (100%).

#### Effect of pH on xylanase stability

For pH stability, the enzyme was incubated with different buffers viz. 50 mM acetate buffer for pH range 4–6, 50 mM phosphate buffer for pH range 6–7, and 50 mM Tris–HCl buffer for pH range 7–10 at 55 °C for 1 h. Thereafter, enzyme activity was determined using the enzyme assay as described above.

#### Effect of metal ions and reagents on activity

The effect of metallic ions at concentration of 5 mM, chelating agents, surfactants, and inhibitors on the activity of crude xylanase were determined by preincubating the enzyme in the presence of Na^+^, Mg^2+^, Ca^2+^, Mn^2+^, Fe^2+^, Zn^2+^, Cu^2+^, K ^+^, Hg^2+^, and Cd^2+^, EDTA (5 Mm), SDS (1%), β-mercaptoethanol (20 mM), and Triton X-100 (1%) for 30 min at 55 °C before adding the substrate (Ozcan et al. 2011). Subsequently, relative xylanase activities were measured at standard enzyme assay conditions. Relative activity was expressed as the percentage of the activity observed in the absence of any compound.

#### Activity of crude enzyme on various carbohydrate substrate

The presence of other carbohydrase was analyzed using oat spelt xylan, birchwood xylan, starch, and CMC (10 mg/ml). The reducing sugar released during the assay was quantified by spectroscopy at *λ*_540_.

#### Effect of organic solvents on xylanase activity

Cell free supernatant having maximum xylanase activity was incubated with 30% (v/v) of different organic solvents, namely, acetone, propanol, ethanol, methanol, chloroform, heptane, cyclohexane, and toluene for 30 min at 55 °C. The residual xylanase activity was estimated against the control, in which solvent was not present.

### Statistical analysis

All determinations were performed at least in three independent replicates, and the control experiment without xylanase was carried out under the same conditions. The experimental results were expressed as the mean of the replicate determinations and standard deviation (mean ± SD). The statistical significance was evaluated using *t* tests for two-sample comparison and one-way analysis of variance (ANOVA) followed by Duncan test. The results were considered statistically significant for *P* values of less than 0.05. The statistical analysis was performed using the R package Version 3.1.1 (Vanderbilt University, USA).

### Nucleotide sequence accession number

The data reported in this work for the nucleotide sequence of the 16S rRNA (1089 bp) gene of the isolate SJ3 have been deposited in the DDBJ/EMBL/GenBank databases under Accession Number KT222887.

## Results and discussion

### Screening of xylanase-producing bacteria from Algerain soil and molecular characterization of the target microorganism

In the current study, ten candidates were obtained from the first screening as xylanase producers. Among them, a bacterium called SJ3, displayed the highest extracellular xylanase activity after 2 days incubation in an initial medium (data not shown) and was, therefore, retained for all subsequent studies.

The physiological and biochemical characteristics of the SJ3 isolate presented in this study were investigated according to well-established protocols and criteria described in the Bergey’s Manual of Systematic Bacteriology as well as the API 50 CHB/E and the API 20E galleries for representative strains. The findings indicated that the SJ3 isolate was Gram-stain-positive, motile, rod-shaped, catalase-positive, aerobic, and endospore forming microorganism. Optimal growth temperature was 37 °C; optimal pH was 7.0. According to the results obtained using the API 50 CHB/E medium and the API 20E strips, the characteristics strongly confirmed that the isolate belongs to *Bacillaceae* order and *Bacillus* genus. The physiological and some biochemical properties of the isolate SJ3 are given in Table 1.

**Table 1:** Morphological, physiological, and some biochemical properties of the isolate *Bacillus oceanisediminis* strain SJ3

Characteristics	*Bacillus oceanisediminis* strain SJ3
Isolation source	Soil
Motility	+
Morphology	Spore forming rods
Gram-stain	+
Temperature for growth	37
Temperature optimum range	25–45
pH for growth	7
pH optimum range	6–9
NaCl for growth (%)	0–12
Indole	−
Methyl red	+
Voges-proskauer	−
catalase	+
Glycerol	+
Erythritol	−
d-Arabinose	−
l-Arabinose	−
Ribose	+
d-Xylose	+
Galactose	+
Glucose	+
Fructose	−
d-Mannose	−
Mannitol	−
Sorbitol	−
Cellobiose	−
Maltose	−
Lactose	−
Saccharose	−
Inulin	−
Strach	−
Gelatin	+

**Fig. 1:**
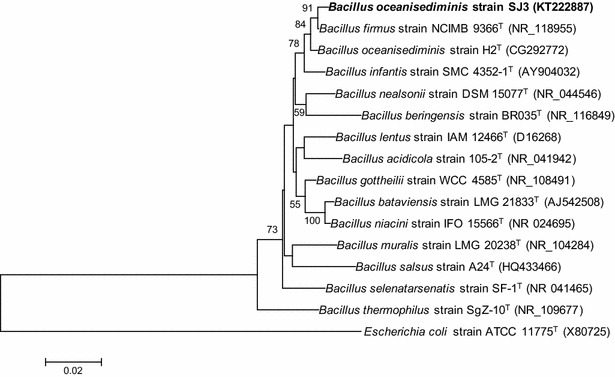
Phylogenetic tree based on 16S rRNA gene sequences showing the position of strain SJ3 within the radiation of the genus *Bacillus*. The sequence of *E. coli* strain ATCC 11775^T^ (Accession No. X80725) was chosen arbitrarily as an outgroup. *Bar* 0.02 nt substitutions per base. Numbers at nodes (>50%) indicate support for the internal branches within the tree obtained by bootstrap analysis (percentages of 100 bootstraps). NCBI accession numbers are presented in *parentheses*

The 16S rRNA gene sequence (KT222887) obtained was submitted to GenBank BLAST search analyses, which yielded a strong homology of up to 99% with those of several cultivated strains of *Bacillus*. From the analysis of the almost-complete 16S rRNA gene sequence, this strain was found to be similar to *B. oceanisediminis* strain H_2_^T^ (99.16% sequence identity). Through the alignment of homologous nucleotide sequence of known bacteria, phylogenetic relationships could be inferred, and the phylogenetic position of the strain and related strains based on the 16S rDNA sequence is shown in Fig. 1. Taken together, the results suggest that this isolate may be assigned as *B. oceanisediminis* strain SJ3.

### Optimization of xylanase production by strain SJ3

**Fig. 2:**
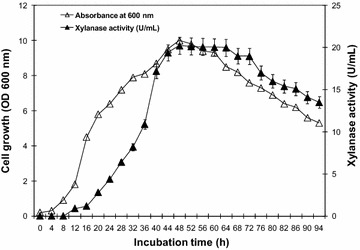
Time course of *Bacillus oceanisediminis* strain SJ3 cell growth (*open diamond*) monitored by measuring the OD at 600 nm and xylanase production (*closed diamond*). *Vertical bars* indicate standard error of the mean (*n* = 3)

In the current study, the bacterial strains were newly isolated from Algerain soil samples (Bejaia north east, Algeria), were screened for their xylanase activities. Using the ratio of the clear zone diameter (onto xylan agar plates) and that of the colony, five isolates exhibiting the highest ratio were tested for xylanase production in liquid culture. Among those strains, a bacterium called strain SJ3, displayed the highest extracellular xylanase activity (20.24 U/ml) after 48 h incubation in an optimized medium (Fig. 2) and was, therefore, retained for all subsequent studies.

Time course of xylanase production showed maximum enzyme activity at 48 h of incubation and thereafter, it remained less constant till 72 h (Fig. 2).

It is the same case with *Bacillus subtilis* strain ASH (Sanghi et al. 2009). The optimum time resulting in maximum enzyme titre is likely to depend on several factors including the microbial strain. A survey of the literature revealed the highest enzyme production from *Bacillus pumilus* strain SV-85S after 36 h (Nagar et al. 2010) and *Bacillus* sp. strain SSP-34 after 96 h (Subramaniyan and Prema 2000) and *B. pumilus* strain VLK-1 after 56 h of incubation (Kumar et al. 2014). In the above reports, the activity of xylanase exhibited a decline after reaching a maximum value, which might be due to proteolysis of the enzyme. However, in the present study, though the incubation period for xylanase production from *B. oceanisediminis* strain SJ3 was shorter than some other *Bacillus* sp. yet it did not decline after attaining the highest level.

### Some biochemical properties of the crude enzyme

Xylanase activity from *B. oceanisediminis* strain SJ3 was efficiently recovered using the TPP technique. A purification fold of 3.48 and a recovery yield of 107% were obtained. Using macroaffinity ligand-facilitated TPP, Sharma and Gupta (2002) purified a xylanase from *Aspergillus niger* with a recovery yield of 60% and a 95-fold purification. The authors reported other recovery parameters using the denatured xylanase and the optimal parameters were 93% and a purification factor of 21 (Roy et al. 2004). TPP has been reported to recover different enzyme activities (e.g., xylanase, cellulase, cellobiase, β-glucosidase, and α-chymotrypsin) from their inactivated/denatured forms (Roy et al. 2004, 2005; Sardar et al. 2007). These findings suggest that TPP may be a valuable technique for the simultaneous renaturation/purification of the multiple enzymes present in a protein mixture. Concerning the high yield recovery obtained in this preliminary study several studies reported high recovery yields (>100%) for the purification of enzymes using the TPP system (Gagaoua and Hafid 2016; Gagaoua et al. 2017).

#### Effect of temperature on xylanase activity

**Fig. 3:**
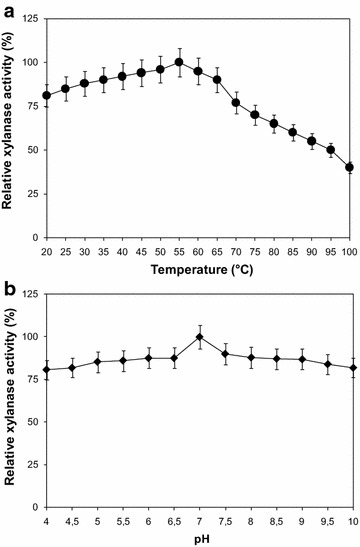
Effects of temperature (**a**) and pH (**b**) on xylanase activity produced by *Bacillus oceanisediminis* strain SJ3 and recovered by three phase partitioning. **a** The enzyme activity was determined by incubating the enzyme with 10 mg/ml oat spelt xylan dissolved in 50 mM phosphate buffer at pH 7. The activity of the enzyme at 55 °C was taken as 100%. **b** The enzyme was incubated at 55 °C with 10 mg/ml oat spelt xylan dissolved in different buffer. Buffer solutions used for pH activity are presented in “Results and discussion”. The activity of the enzyme at pH 7.0 was taken as 100%. Each point represents the mean of three independent experiments. Vertical bars indicate standard error of the mean (*n* = 3)

The effect of temperature on the xylanase activity from *B. oceanisediminis* strain SJ3 is shown in Fig. 3a, for 10 min reaction the optimum temperature was 55 °C (assayed in the range 20–100 °C), the xylanase produced by *Bacillus brevis* is also optimally active at the same temperature (Goswami et al. 2013). The optimum temperature of the enzyme is near to that of the xylanases from *B. subtilis* strain CXJZ isolated from the degumming line (60 °C) (Guo et al. 2012) and *Bacillus* sp. strain 41M-1 which showed maximum activity at 50 °C (Nakamura et al. 1995) and *Bacillus* sp. strain BP-23 (50 °C) (Blanco et al. 1995) but distant from that of the xylanases produced by *Bacillus halodurans* strain TSEV_1_ (80 °C) (Kumar and Satyanarayana 2014), *Caldicoprobacter algeriensis* strain TH7C1^T^ (Bouacem et al. 2014), *B. subtilis* strain GN156 (40 °C) (Pratumteep et al. 2010), and *Bacillus amyloliquefaciens* strain CH51 (25 °C) (Baek et al. 2012).

#### Effect of pH on xylanase activity

The optimum pH of *B. oceanisediminis* strain SJ3 xylanase activity (assayed in the range 4–10) is 7 (Fig. 3b). Other xylanases from *Bacillus* strains so far characterized generally show wide differences in their optimal pH, going from acidic values, such as 4 for the glycosyl hydrolase family 11 xylanase from *B. amyloliquefaciens* strain CH51 (Baek et al. 2012), 5 for the xylanase activity produced by *B. subtilis* strain GN156 (Pratumteep et al. 2010), 5.8 for the xylanase from *B. subtilis* strain CXJZ (Gang et al. 2012), up to 9 in the case of the endoxylanase activity from *B. halodurans* strain TSEV_1_ (Kumar and Satyanarayana 2013, 2014).

#### Thermostability profile of the xylanase activity

**Fig. 4:**
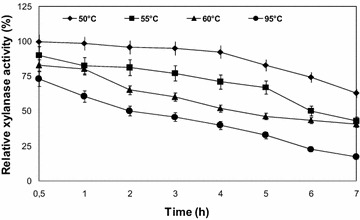
Thermostability profile of *Bacillus oceanisediminis* strain SJ3 xylanase at pH 7 at different temperatures. (*closed diamond*): 50 °C, (*closed square*): 55 °C, (*closed triangle*): 60 °C, (*closed circle*): 95 °C. Samples were taken at 1 h interval and relative activity was determined. The activity of the non-heated enzyme was taken to be 100%. Each point represents the mean of three independent experiments. *Vertical bars* indicate standard error of the mean (*n* = 3)

Thermal stability was carried out by preincubating xylanase up to 7 h at 50, 55, 60, and 95 °C (Fig. 4), at 50 °C there was no significant decrease in xylanase activity during 4 h. The enzyme was stable at 50 °C, with a half-life time of 9 h, a half-life time of 6 and 4.72 h was respectively observed at 55 and 60 °C. *B. brevis* xylanase is less thermostable, it showed a half-life time of 3 h at 55 °C (Goswami et al. 2013).

At 95 °C the profile obtained for thermostability showed that 50% of the original activity was retained after 2 h exposure, the results clearly indicated that the suitable temperature range for industrial application for xylanase from *B. oceanisediminis* strain SJ3 was 50–95 °C. This xylanase is more thermostable than *B. amyloliquefaciens* strain XR44A xylanase activity which showed a half-life time of 5 min at 70 °C, 15 min at both 50 and 60 °C, and 2 h at 40 °C. Interestingly, it retained 90% of activity for at least 2 days at 30 °C, with a half-life time of 7 days. The enzyme immediately loses activity at temperatures higher than 70 °C (Amore et al. 2015), the xylanase produced by *Bacillus aerophilus* strain KGJ2, retained more than 90% activity after incubation at 80–90 °C for 60 min (Gowdhaman et al. 2014). The enzyme produced by *Bacillus* sp. strain DM-15 was stable for 15 min at 60 °C while 95% of the original activity was lost at 90 °C (Ozcan et al. 2011).

The xylanase from *Pseudomonas macquariensis* had half-life time of 2 h at 50 °C whereas it had a half-life time of 1 h at 60 °C. At high temperatures, enzyme gets partly unfolded (Sharma et al. 2008). The xylanase of *B. oceanisediminis* strain SJ3 is highly thermostable, such enzymes with high thermostability and an ability to function at wide pH range are desirable for many industrial processes which take place at very high or low pH and high temperature. With this respect, the strain could be a good source for industrial and biotechnological applications.

#### pH stability profile of the xylanase activity

**Fig. 5:**
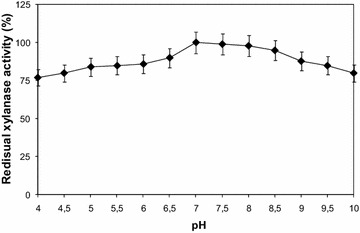
pH stability of the xylanase activity produced by *Bacillus oceanisediminis* strain SJ3 and recovered by three phase partitioning. The crude enzyme was incubated with 50 mM buffers at 55 °C for 1 h and relative activity was measured under the standard assay conditions. The activity of the enzyme at optimum pH was taken as 100%. Buffer solutions used for pH stability are presented in “Results and discussion”. Each point represents the mean of three independent experiments. *Vertical bars* indicate standard error of the mean (*n* = 3)

It is observed that the highest xylanase activity was established at pH 7.0; on the other hand, it was found to be most stable at pH 7.0–8.0 but it was also stable in a range of pH 5–10 and at pH 10 approximately 80% of its activity was retained (Fig. 5). The enzyme stable in alkaline conditions were characterized by a decreased number of acidic residues and an increased number of arginines (Hakulinen et al. 2003). The similar pattern of pH stability was also found in *Bacillus vallismortis* strain RSPP-15 (Gaur et al. 2015).

#### Effect of metallic ions, reagents, and inhibitors on xylanase activity

We investigated the effects of metallic ions and other reagents on the activities of the crude xylanase (Table 2). Most of the metallic ions (at concentration of 5 mM) tested had little influence on the activity, the same results were obtained with the xylanases produced by *Bacillus* sp. strain SPS-0 (Bataillon et al. 2000); in this experiment, maximum xylanase production was reported in the presence of Ca^2+^ (138%); some other researchers also reported that Ca^2+^ ion strongly stimulated xylanase activity. Slightly stimulation was also observed by addition of Mg^2+^ (106%) (Mamo et al. 2006; Lv et al. 2008; Ozcan et al. 2011).

**Table 2:** Effect of different metallic ions, surfactants, chelating agents, and inhibitors on xylanase activity

Chemical additives	Concentration	Relative enzyme activity (%)^a^
Control	–	100 ± 2.5
Mg^2+^ (MgCl_2_)	5 mM	106 ± 2.6
Ca^2+^ (CaCl_2_)	5 mM	138 ± 4.1
Fe^2+^ (FeSO_4_)	5 mM	88 ± 2.2
K^+^ (KCl)	5 mM	98 ± 2.4
Cu^2+^ (CuCl_2_)	5 mM	92 ± 2.3
Na^+^ (NaCl)	5 mM	94 ± 2.3
Mn^2+^ (MnCl_2_)	5 mM	99 ± 2.5
Cd^2+^ (CdCl_2_)	5 mM	83 ± 2.0
Zn^2+^ (ZnCl_2_)	5 mM	95 ± 2.3
Hg^2+^ (HgCl_2_)	5 mM	20 ± 0.6
Triton X-100	1%	93 ± 2.3
SDS	1%	87 ± 2.2
EDTA	5 mM	88 ± 2.2
β-Mercaptoethanol	20 mM	86 ± 2.2

Xylanase activity measured in the absence of any chemical additives was taken as control (100%). The non-treated and dialyzed enzyme was considered as 100% for metallic ion assay. Residual activity was measured at pH 7.0 and 55 °C

^a^Values represent means of three independent replicates, and ±standard errors are reported

On the other hand, the inhibition of xylanases by calcuim and magnesium ions have also been reported (Hmida-Sayari et al. 2012; Chang et al. 2017). Xylanase was strongly inhibited in the presence of Hg^2+^. Similar results were observed in case of *B. subtilis* (Sanghi et al. 2010) and *Bacillus halodurans* strain PPKS-2 (Prakash et al. 2012), it has been reported that the xylanase activity was inhibited by mercury ion, which might be due to its interaction with sulfhydryl groups of cysteine residue in or close to the active site of the enzyme (Bastawde 1992).

The chelating agent EDTA enveloping metal ions extensively did not change the xylanase activity (Table 2) that means the enzyme did not require metal ions for its catalysis.

Triton X-100 and β-mercaptoethanol had little effect on the xylanase activity (Table 2) whereas the *Bacillus* DM-15 xylanase is sensitive (Ozcan et al. 2011).

Total inactivation due to SDS has already been reported for xylanases of different origins (Fujimoto et al. 1995), in contrast to the resistance to SDS was found in this study, with 87% relative activity after 10 min at 55 °C (Table 2).

### Activity of the crude xylanase on various carbohydrate substrates

**Fig. 6:**
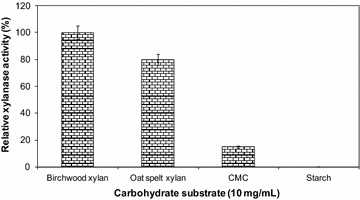
The effect of the carbohydrate substrate source on the xylanase activity produced by *Bacillus oceanisediminis* strain SJ3 and recovered by three phase partitioning. The enzyme was incubated with 10 mg/ml of substrate at 55 °C and pH 7.0. Each point represents the mean of three independent experiments. *Vertical bars* indicate standard error of the mean (*n* = 3)

Activity of the crude enzyme on some carbohydrate was showed at Fig. 6, the crude enzyme mainly contained xylanase as indicated by the highest activity on birchwood xylan (25 U/ml) and oat spelt xylan (20 U/ml). The crude enzyme did not contain amylase but hardly cellulase (1.99 U/ml). Crude enzymes produced by *Bacillus* sp. strain AQ1 not only showed xylanolytic activity but also amylolytic and cellulolytic activity (Wahyuntari et al. 2009). Comparisons to the large literature studies as summarized in Table 3.

**Table 3:** Production of xylanases from various bacteria, namely *Bacillus* genus and comparisons to our findings

Organism	Substrate	Cultivation conditions (temperature and pH)	Xylanase activity (U/ml or U/mg)	References
*Bacillus oceanisediminis SJ3*	Oat spelt xylan	55 °C; pH 7.0	20.24 U/ml	Present study
*Jonesia denitrificans BN*-*13*	*Birchwood xylan*	50 °C; pH 7.0	77 U/mg	Boucherba et al. (2014)
*Bacillus pumilus* MTCC 8964	Oat spelt xylan	60 °C; pH 6	241 U/ml	Kumar et al. (2010)
*Bacillus brevis*	Birchwood xylan	55 °C; pH 7.0	1.52 U/ml	Goswami et al. (2013)
*Bacillus brevis*	*wheat straw*	55 °C; pH 7.0	4380 U/mg	Goswami et al. (2014)
*Bacillus* sp. strain BP-23	Birchwood xylan	50 °C; pH 5.5	40.2 U/mg	Blanco et al. (1995)
*Bacillus halodurans* TSEV_1_	*Cane molasses*	80 °C; pH 9.0	15 U/ml	Kumar and Satyanarayana (2014)
*Bacillus amyloliquefaciens* CH51	Birchwood xylan	25 °C; pH 4.0	701.1 U/mg	Baek et al. (2012)
*Bacillus subtilis* CXJZ	birchwood and oat spelt xylan	60 °C; pH 5.8	36,633 U/mg	Gang et al. (2012)
*Bacillus pumilus* SSP-34	Oat spelts xylan	50 °C; pH 6.0	1723 U/mg	Subramaniyan (2012)
*Bacillus pumilus* SV-205	Wheat bran	60 °C; pH 10.0	7382.7 U/ml	Nagar et al. (2012)
*Bacillus subtilus* BS05	Sugarcane bagasse	50 °C; pH 5.0	17.58 U/ml	Irfan et al. (2012)
*Gracilibacillus* sp. TSCPVG	Birchwood xylan	pH 7.5	1667 U/mg	Poosarla and Chandra (2014)
*Paenibacillus* sp. NF1	Oat spelt xylan	60 °C; pH 6.0	3081.05 U/mg	Zheng et al. (2014)
*Paenibacillus macerans* IIPSP3	Beechwood xylan	60 °C; pH 4.5	4170 U/mg	Dheeran et al. (2012)
*Anoxybacillus* flavithermus TWXYL3	Oat spelt xylan	65 °C; pH 6.0 and pH 8.0	117.64 U/mg	Ellis and Magnuson (2012)

Based on the available data from this experiment, the difference in crude enzyme on the different xylan substrate could not be explained yet. It is still needed more complete studies to elaborate the type of xylanolytic activities present in the crude enzyme of *B. oceanisediminis* strain SJ3. From preliminary study, it can be observed that the strain SJ3 was able to grow and produce xylanases using commercial xylan. The pH and temperature optima of the preparation were 7 and 55 °C, respectively, and the enzyme was stable in a range of pH 5–10 retained 50% of its activity during 6 h at 55 °C. The enzyme is also resistant to hydrophobic solvents, these properties place this enzyme as promising for industrial and biotechnological applications especially lignocellulose bioconversion and bioethanol production.

### Effect of organic solvents of the xylanase activity

**Fig. 7:**
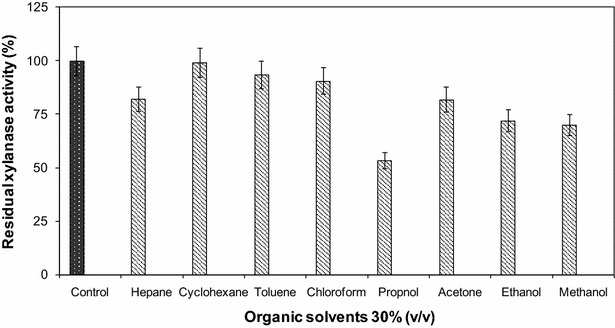
Effect of organic solvents on xylanase activity produced by *Bacillus oceanisediminis* strain SJ3 and recovered by three phase partitioning. Relative xylanase activity was expressed as a percentage of the control reaction without solvent. Each point represents the mean of three independent experiments. *Vertical bars* indicate standard error of the mean (*n* = 3)

The xylanase from *B. oceanisediminis* strain SJ3 is resistant to hydrophobic solvents: heptan, chloroform, toluene, and cyclohexane (the relative activity is 99.2%) but a loss of the enzyme activity was observed by addition of 30% (v/v) of methanol, ethanol, propanol, and acetone (Fig. 7). These alcohols completely inhibited the enzyme from *Termitomyces* sp. and *Macrotermes subhyalinus* at 30% (v/v) and 60% (v/v), respectively. Primary alcohols including methanol, ethanol, and isopropanol as well as polyhydric alcohol containing glycol and glycerol, all showed inhibitory effects on *A. niger* strain C3486 xylanase activity which retained around 90% at the concentration of 2% (v/v) and less than 60% of its initial activity at 30% (Yang et al. 2010).

In some cases, the presence of solvents enhanced the xylanase activity, for example the xylanase of *B. vallismortis* is extraordinarily stable in the presence of all organic solvents under study. After incubation with *n*-dodecane, isooctane, *n*-decane, xylene, toluene, *n*-hexane, *n*-butanol, and cyclohexane, the xylanase activity increased to 230.8, 137.7, 219.8, 107, 190.5, 194.7, 179.3, and 111.6%, respectively (Gaur and Tiwari 2015).

## Conclusion

In conclusion, a new extracellular thermostable xylanase from *B. oceanisediminis* strain SJ3 was produced and characterized in this study. The preliminary results of the use of Three phase partitioning for the recovery of the xylanase were presented. The time course for xylanase accumulation by strain SJ3 in xylan-based medium showed that the highest xylanase activity reached 20.24 U/ml in an optimized medium with oats spelt xylan used as a substrate after 48 h of cultivation. The crude xylanase from strain SJ3 was biochemically characterized. The results revealed that the enzyme was highly stable and active at high temperature (55 °C) and alkaline pH 7.0. Properties of this enzyme such as high specific activity, wide range of pH optimum and stability, and thermostability at elevated temperature as well as organic solvents tolerance, are appropriate for industrial and biotechnological applications. Interestingly, this enzyme presented high xylanolytic activity with oats spelt xylan, and was very effective in the pulp bleaching industry, thus offering a potential promising candidate for application in biotechnological bioprocesses. Accordingly, further studies, some of which are currently underway, are needed to investigate the purification to homogeneity and encoding gene, perform site-directed mutagenesis, and determine its structure–function relationships.
